# Elevated Baseline Salivary Protease Activity May Predict the Steadiness of Gingival Inflammation During Periodontal Healing: A 12-Week Follow-Up Study on Adults

**DOI:** 10.3390/pathogens9090751

**Published:** 2020-09-15

**Authors:** Ulvi Kahraman Gürsoy, Dareen Fteita, Floris J. Bikker, Maria Anastasia Grande, Kamran Nazmi, Mervi Gürsoy, Eija Könönen, Daniel Belstrøm

**Affiliations:** 1 Department of Periodontology, Institute of Dentistry, University of Turku, 20520 Turku, Finland; dareen.fteita@utu.fi (D.F.); mervi.gursoy@utu.fi (M.G.); eija.kononen@utu.fi (E.K.); 2 Department of Oral Biochemistry, Academic Centre for Dentistry Amsterdam, Free University and University of Amsterdam, 1081LA Amsterdam, The Netherlands; f.bikker@acta.nl (F.J.B.); k.nazmi@acta.nl (K.N.); 3 Section for Clinical Oral Microbiology, Periodontology, Department of Odontology, Faculty of Health and Medical Sciences, University of Copenhagen, DK-2200 Copenhagen, Denmark; mgra@sund.ku.dk (M.A.G.); dbel@sund.ku.dk (D.B.)

**Keywords:** chemokine CCL22, macrophage inflammatory proteins, periodontitis, *Porphyromonas gingivalis*, saliva

## Abstract

Aim was to profile salivary total protease, *Porphyromonas gingivalis* gingipain, and neutrophil elastase activities in relation to the resolution of periodontal inflammation, salivary macrophage-derived chemokine (MDC), and macrophage inflammatory protein-1α concentrations. Nonsurgical periodontal treatment was performed in 24 periodontitis patients in a prospective interventional study design. Periodontal clinical parameters were recorded, and stimulated saliva samples were collected at baseline and 2, 6, and 12 weeks after treatment. Salivary total protease and gingipain activities were determined using fluorogenic substrates, elastase activity by chromogenic substrates, and cytokine concentrations by Luminex immunoassay. For statistical analyses, generalized linear mixed models for repeated measures were used. Salivary total protease activity elevated, while gingival inflammation and plaque accumulation decreased 2 and 6 weeks after periodontal therapy. Salivary MDC concentration was elevated 12 weeks after periodontal treatment. Patients with elevated protease activities at baseline in comparison to patients with low baseline total protease activities, had higher levels of gingival inflammation before and after periodontal treatment. In conclusion, elevations in salivary total protease activity seem to be part of periodontal healing at its early phases. Higher levels of salivary total protease activities before periodontal treatment may predict the severity and steadiness of unresolved gingival inflammation.

## 1. Introduction

Periodontitis is characterized by a complex cascade of inflammatory interactions between the periodontal pathogenic bacteria residing in the subgingival biofilm and the host immune system. Periodontal pathogens produce proteolytic enzymes that impair the host response and epithelial integrity, which, in turn, facilitate microbial invasion into the surrounding tissues [1,2]. The subsequent recruitment and migration of neutrophils to the site of infection require the activation of a chemotactic inflammatory network [3,4]. Besides their antimicrobial activity, neutrophils constitute a main source of host proteolytic enzymes, including neutrophil elastase, matrix metalloproteinases, cathepsins, and serine proteases, which collectively contribute to the degradation of periodontal tissues [5]. The migration of inflammatory cells towards the wound site is dominated by the mesenchymal cell differentiation of the phagocytic neutrophils, monocytes, and lymphocytes, which engulf invading microbes and secrete proteolytic enzymes to digest necrotic tissues. Furthermore, macrophages and lymphocytes orchestrate the formation of granulation tissue through the secretion of growth factors and cytokines, which, subsequently, recruit resident cells to establish a cell-rich remodeling tissue [6].

On one hand, the initiation of cytokine signaling by tissue resident cells and neutrophils induces macrophage polarization and T-cell accumulation in periodontal tissues, which results in the resolution of inflammation and wound healing [7]. On the other hand, bacterial proteases can inhibit the production of several chemokines, phagocytic activity of macrophages, and neutrophil chemotaxis, leading to delayed wound healing [8,9]. Precisely, *Porphyromonas gingivalis*, a major periodontitis-associated pathogen, has two trypsin-like cysteine proteases (arginine-specific gingipain R and lysine-specific gingipain K) [10]. The two extracellular gingipains demonstrate independent catalytic domains separated from the activities of their binding domains, e.g., hemagglutination and epithelial adhesion via hemagglutinin/adhesin domains and coagulation action through the kallikrein/kinin domains [11]. Immunologically, gingipains induce neutrophil dysfunction, complement pathway impairment, and cleavage of immunoglobulins [2,12]. To enable a persistent colonization of *P. gingivalis*, gingipains degrade macrophage (CD14) through the inactivation of lipopolysaccharide receptors on the leukocytes surface [2,13]. An in vitro wound-healing model revealed that gingipains R and K may significantly influence the capability of *P. gingivalis* to inhibit the oral epithelial cell migration in a scratched epithelial layer [14]. Indeed, gingipain can activate periodontal resident cells (epithelial cells and fibroblasts) to become a collagen-degrading phenotype and impair tissue regeneration [15]. Taken together, it is possible to hypothesize that clinically elevated *P. gingivalis* gingipain levels cause a disruption in matrix remodeling and stimulate delayed wound healing after the treatment of periodontitis.

The ultimate goal in periodontal diagnostics is to monitor the health status, detect the periodontal disease initiation, and predict the successful treatment outcome. As an easily collected and noninvasive specimen, saliva has been used as a diagnostic fluid in medicine [16]. General aims in periodontal research on salivary diagnostics are to find marker(s) that could be used, preferably as chairside tests, e.g., to determine the activity of periodontitis or the outcome of the treatment, or, to a lesser extent, to detect periodontitis in field studies. Elevated levels of proteolytic enzymes (*P. gingivalis* gingipain, human neutrophil elastase, matrix metalloproteinases, total proteolytic activity), cytokines (interleukin-1β), and chemokines (MDC, MIP-1α, MCP-2) have been found in tissue and saliva samples of periodontitis patients in comparison to their controls [16,17,18,19,20,21]. Here, we hypothesized that elevated salivary protease activity of both host and bacterial origin are related to delayed tissue healing after periodontal treatment, thus can act as predictive indicator of treatment outcome. Therefore, the aim of this study was to profile the total protease, gingipain, and elastase activities in saliva of periodontitis patients before and after treatment and to examine whether their levels are related to clinical periodontal indices, salivary macrophage-derived chemokine (MDC), and macrophage inflammatory protein (MIP)-1α concentrations. Furthermore, it was also to examine whether the abundance of *P. gingivalis* counts affects the resolution of periodontal inflammation. 

## 2. Results

Six participants dropped out during the follow-up and, additionally, the salivary samples of one patient were lost during laboratory processing. A total of 96 saliva samples from 24 participants were biochemically analyzed.

Clinical parameters responded positively to nonsurgical periodontal treatment. Detailed description of the changes in bleeding on probing (BOP), plaque index (PI), probing depth (PD), and clinical attachment level (CAL) scores during the follow-up were published previously [20,22]. In brief, the following clinical measurement scores were recorded during the baseline and periodontal healing: PI: 84.2% (baseline), 41% (week 2), 43% (week 6), and 41.8% (week 12); BOP: 56% (baseline), 27.1% (week 2), 34.6% (week 6), 41.3% (week 12); and PD: 3.4 mm (baseline), 3.0 mm (week 12). CAL: 4.1 mm (baseline), 3.7 mm (week 12). Mean PD score of the deepest 4 sites at baseline was 6.4 (range: 5.0–8.8) and 12 weeks after the periodontal treatment was 5.0 (range: 3.3–7.3), whereas mean CAL score of the same sites at baseline was 7.0 (range: 5.5–9.3) and 12 weeks after the periodontal treatment was 5.7 (range 3.3–8.8), (*p* < 0.001).

**Figure 1:**
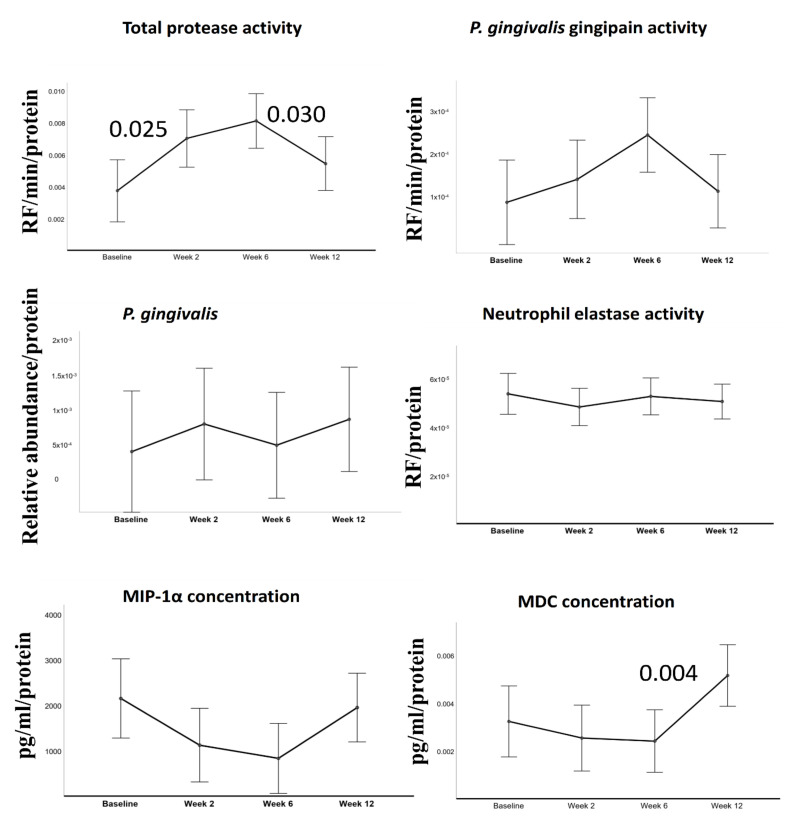
Estimated means of salivary parameters. In all tests, bleeding on probing (BOP) was included into the model as a covariant. All units are presented as per protein values. Error bars indicate 95% confidence intervals. *P* values above the connector lines indicate significant difference between visits.

Total protease, gingipain, neutrophil elastase activities, *P. gingivalis* relative abundance, and MCP and MIP-1α concentrations before and after periodontal treatment are all given in Figure 1.

All data are presented in relation to salivary protein levels. BOP % was included in the model as a time-varying covariate. Generalized linear mixed models for repeated measures demonstrated significant changes in total protease activity (F = 3.712, *p* = 0.014) and MDC concentrations (F = 3.685, *p* = 0.015) over time. After periodontal treatment, there was a slight increase in gingipain activity after periodontal treatment, but the observed changes were not statistically significant (F = 2.241, *p* = 0.089), while total protease activity elevated up to week 6 (*p* = 0.025) and dropped to baseline levels at week 12. Salivary MDC concentrations stayed steady at weeks 2 and 6 but elevated significantly at week 12 (*p* = 0.004). 

**Figure 2:**
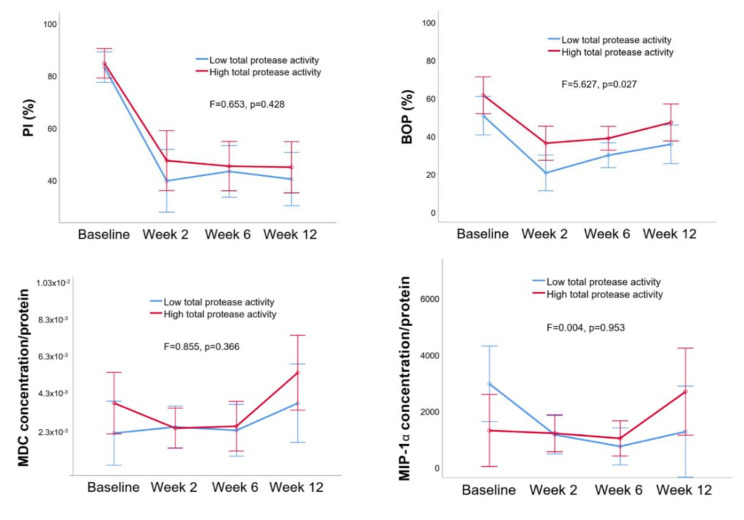
Estimated means of plaque index (PI) (%) and BOP (%) and macrophage inflammatory protein (MIP)-1α and macrophage-derived chemokine (MDC) concentrations (per protein) in low and high total protease activity groups. Error bars indicate 95% confidence intervals. Between group differences are given as F and *p* values (*p* < 0.05 indicates a significant difference).

Based on the activity of proteases and gingipain at baseline, the patients were divided into the low and high total protease activity groups. There were significant differences in BOP percentages over time between the low and high total protease activity groups (Figure 2, *p* = 0.014). 

**Figure 3:**
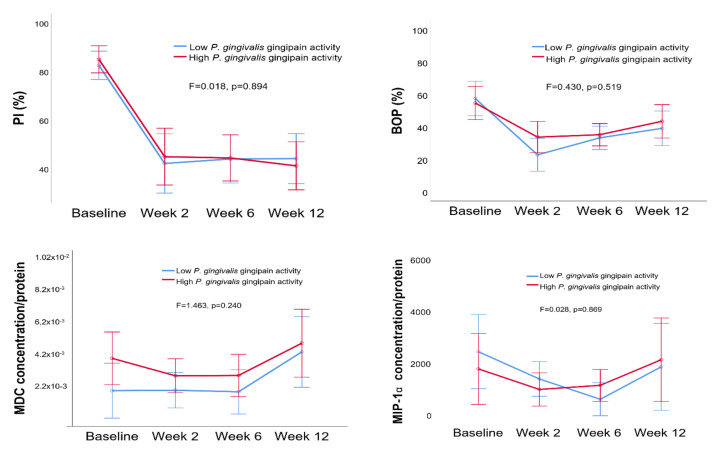
Estimated means of PI (%) and BOP (%) and MIP-1α and MDC concentrations (per protein) in low and high *P. gingivalis* gingipain activity groups. Error bars indicate 95% confidence intervals. Between group differences are given as F and *p* values (*p* < 0.05 indicates a significant difference).

**Figure 4:**
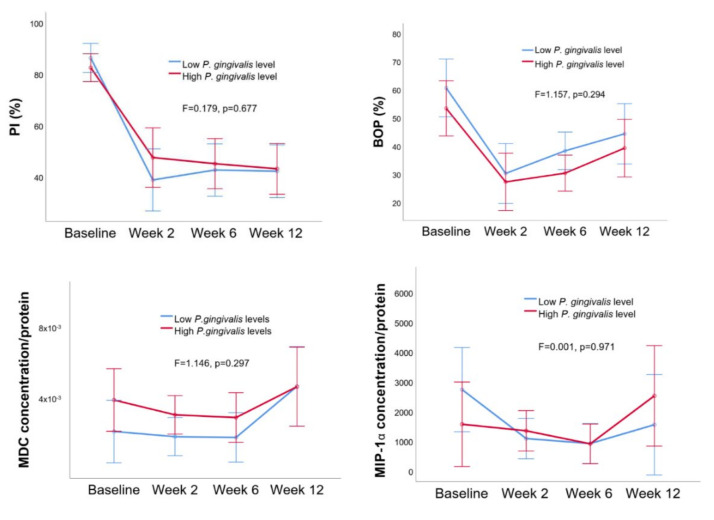
Estimated means of PI (%) and BOP (%) and MIP-1α and MDC concentrations (per protein) in low and high *P. gingivalis* relative abundance groups. Error bars indicate 95% confidence intervals. Between group differences are given as F and *p* values (*p* < 0.05 indicates a significant difference).

No difference was observed in PI, BOP, MCP, or MIP-1a levels between the low and high *P. gingivalis* gingipain activity groups (Figure 3) or between the low and high *P. gingivalis* relative abundance groups (Figure 4).

## 3. Discussion

After periodontal treatment, the elevation in salivary total protease activity seems to be part of the healing response at its early phases (during 2–6 weeks). Moreover, we showed for the first time that high pretreatment salivary protease activity is related to the limited post-treatment resolution of gingival inflammation.

The main strength of this study was its follow-up design, where bias was minimized by performing all laboratory and statistical analyses blindly. The extension and severity of gingival inflammation, estimated during BOP recordings, varies with time. To eliminate the effect of clinical inflammation on salivary cytokine concentrations, BOP levels were included in the generalized linear mixed model as time-dependent variant. Periodontal treatment and follow-up were performed according to current clinical guidelines at the Department of Odontology, University of Copenhagen. Accordingly, follow-up visits after 2 and 6 weeks are performed to evaluate self-performed oral hygiene procedures, whereas the clinical effect of the performed nonsurgical treatment is evaluated at the 12 weeks recall visit. Treatment is expected to have immediate impact on PI and BOP, which is why these were already recorded after 2 weeks. On the other hand, substantial changes in PD and CAL are not expected before after 12 weeks. In the present study, elastase levels were analyzed to define the neutrophil activity, and MIP-1α were analyzed to identify the macrophage levels. However, as we could not measure the activities of all salivary proteases individually, it was not possible for us to determine the identity of the proteases that were elevated after periodontal treatment. Due to the limited amount of sample material, salivary activities of several major host proteases, like matrix metalloproteinases and cathepsins, were not determined. This needs to be taken as a limitation of this study. Finally, the sample size was estimated using longitudinal data on α-diversity of the salivary microbiota as previously described [22]. In the present continuation study, we performed additional analysis on already collected salivary samples. Accordingly, we did not perform a new sample size estimation on the parameters measured in the present study. 

In injured human periodontal ligaments, host-derived cysteine and serine proteases such as cathepsins and elastases are sequentially expressed as a response to inflammation or microbial invasion. These proteases function as inducers of mesenchymal proliferation and epithelial cell migration and, thus, stimulate wound closure [23]. Concerning other host-derived proteases, similar findings have shown that matrix metalloproteinases contribute to the re-epithelialization process during wound healing, and their inappropriate expression (dose-dependent) may lead to significant impairment in the healing process [24,25]. Reduced salivary trypsin-like protease, elastase-like protease, and general proteases have been demonstrated 8 months after periodontal treatment [21]. Since our follow-up period was only for 12 weeks, this may explain why we could not observe changes in elastase activities.

A recent experimental gingivitis study showed that the time required to develop gingival inflammation as a response to plaque accumulation shortens significantly when salivary total protease activity elevates [18]. In line with these findings, our results indicate that periodontitis patients with high total protease activity have steady gingival inflammation after periodontal treatment. Such a difference was not observed between the groups of high and low gingipain activity or high and low *P. gingivalis* relative abundance. To the best of our knowledge, changes in total salivary protease activity have not been previously analyzed in saliva samples collected from periodontitis patients before and after treatment. However, when the activity of individual enzymes was measured, activities of MMP-8 and elastase were found to decrease after periodontal treatment [26]. Nevertheless, there is one experimental gingivitis study, which showed that the salivary total protease activity was elevated even after the resolution of gingivitis [18]. Within the limitations of our study, it was not possible to define exactly which proteases were behind the increase in total enzyme activities.

MDC is a strong chemoattractant for Th2 lymphocytes [27]. According to our findings, salivary MDC concentrations elevated at 12 weeks after periodontal treatment, indicating a late recruitment of Th2 cells in periodontal healing. Another explanation of steady salivary concentrations of MDC and MIP-1α at 2 and 6 weeks after periodontal therapy observed in our study can be related to elevated protease activities. In periodontitis, MIP-1α is induced by macrophages and infected keratinocytes, exhibiting higher expression levels in relation to the severity of inflammation [8,28,29]. As an inflammatory cell-recruiting chemokine, MIP-1α attracts first cytotoxic T cells, and at a later stage, directs B-lymphocytes to wounded tissues in order to induce an acute inflammatory zone [8]. In addition to MIP-1α, as shown in a recent follow-up study on nonsurgical treatment of periodontitis patients, MDC levels in saliva correlate with the abundance of specific periodontitis-related bacteria, reflecting the disease stage and predicted treatment outcomes [20]. Steady concentrations of salivary MIP-1α have been detected at baseline and 16 weeks after periodontal treatment [30], which was also observed in our study. 

It has been demonstrated that *P. gingivalis* is able to subvert host immune response by degrading chemokines with its produced gingipains [31]. In addition, cathepsins can specifically degrade or inactivate several cytokines and chemokines, including MIP-1α [32]. Therefore, it is possible that the elevation in salivary protease activity after periodontal treatment causes degradation of salivary chemokines, which eventually may lead to misinterpretations in chemokine expression levels. 

In conclusion, observed elevations in salivary total protease activity are part of early periodontal healing. High levels of salivary total protease activity before periodontal therapy may predict the limited resolution of gingival inflammation.

## 4. Materials and Methods

This research work is the continuation of a recently published study series on periodontal healing, using salivary samples of the same study population [20,22]. The recruitment of periodontitis patients, their treatments with clinical recordings, and saliva sample and data collections were performed at the Department of Odontology, University of Copenhagen, from September 2016 to the beginning of January 2017. The study protocols were reported to the Danish Data Authority (SUND-2016-58) and accepted by the regional ethical committee of the capital region of Denmark (H- 16016368). This study was registered at clinicaltrials.gov (NCT02913248). All participants signed written informed consent before participation.

The sample size was estimated using longitudinal data on α-diversity of the salivary microbiota [17]. Patients seeking periodontal treatment at the Department of Odontology, University of Copenhagen, who fulfilled the inclusion criteria of the present study, were consecutively enrolled until the adequate sample size was reached.

### 4.1. Study Population

Thirty-one patients diagnosed with moderate to severe periodontitis were included in the study. The patient recruitment protocols have been described in detail previously [22]. Briefly, all study participants were of Caucasian ethnicity, had at least 20 teeth, with an age range of 47–75. Exclusion criteria were: presence of systemic diseases or hyposalivation, used antibiotics or medications with known effects on the periodontium, or having received periodontal treatment during the preceding 3 months prior to the initiation of the study.

### 4.2. Clinical Examination and Periodontal Treatment

The same clinician (M.A.G.) performed periodontal treatments and clinical measurements. As the study population was recruited and all samples were collected during 2016–2017, periodontitis was diagnosed and classified according to the American Academy of Periodontology [33]. Thus, periodontitis was defined as follows: ≥4 teeth with BOP, PPD ≥5 mm, and CAL and radiographic bone loss ≥3 mm. However, when the new 2017 classification of periodontal diseases was applied, all participants were diagnosed as periodontitis and categorized in stage two or three (moderate to severe periodontitis)

Before treatment, PD, CAL, PI, and BOP were measured at six sites per tooth. At baseline, patients received full-mouth nonsurgical periodontal treatment, that is, scaling and root planning with hand and ultrasonic instruments. Furthermore, detailed oral hygiene instructions were given at baseline and at follow-up visits at 2 and 6 weeks after treatment. No antibiotic treatment was administered. After periodontal treatment, BOP and PI measurements were repeated at week 2, 6, and 12, and PI and CAL measurements were repeated at week 12. 

### 4.3. Saliva Collection

A minimum of 2 mL paraffin-stimulated saliva sample was collected from each patient before and 2, 6, and 12 weeks after periodontal treatment [20]. Saliva collections were performed between 8 a.m. and 3 p.m. by the same clinician (M.A.G.), and saliva samples were immediately frozen and stored at −80 °C until sent to Institute of Dentistry laboratories of University of Turku for biochemical analyses.

### 4.4. Analyses of P. gingivalis Gingipain, Total Protease, and Neutrophil Elastase Activities

Saliva samples were thawed and centrifuged at 9300× *g* for 5 min at room temperature. Supernatants were used in enzyme activity analyses. *P. gingivalis*-specific gingipain activity was determined by using the gingipain-reactive fluorogenic substrate FITC-Ahx-(L)Arg-(L)Arg-KDbc) [19,34,35]. Total protease activity was determined using a broad-spectrum fluorogenic substrate, PEK-054 [18,19,36]. Protease activity was defined in relative fluorescence activity per min (RF/min) [35,36]. Neutrophil elastase activity was measured by using a chromogenic substrate (s4760, Sigma Aldrich, Espoo, Finland) according to the manufacturer’s instructions.

### 4.5. Analyses of Salivary P. gingivalis Relative Abundance and MIP-1α and MDC Concentrations

*P. gingivalis* relative abundance, MIP-1α and MDC concentrations, and the detection methods applied in the present study have been described in detail elsewhere [20,22]. Initially, bacterial DNA was isolated with MagNA Pure 96 (Roche, Mannheim, Germany). The Human Oral Microbe Identification using Next Generation Technique (HOMINGS) was utilized to sequence the V3-V4 region of the 16S rDNA. Finally, generated sequences were annotated with 692 reference sequences retrieved from the Human Oral Microbe Database (HOMD) using the customized software Probe-seq developed at Forsyth Institute, Boston, USA. MDC and MIP-1α were identified in supernatants using the Luminex xMAP technique (Luminex Corporation, Austin, TX, USA) with optimized commercial kits (Pro-human cytokine group 1 assays; Bio-Rad, Santa Rosa, CA, USA) according to the manufacturer’s protocol. The limit of detection (LOD) of the assay was 0.5 pg/mL for MDC and 0.3 pg/mL for MIP-1α.

### 4.6. Analysis of Salivary Protein Levels

Protein concentrations of each saliva sample were determined by the Bradford method (Bio-Rad Laboratories Inc.) as described by Hammond and Kruger, 1988 [37].

### 4.7. Statistical Analysis

Generalized linear mixed models for repeated measures were utilized to examine changes in the salivary variables (*P. gingivalis* gingipain, total protease, and neutrophil elastase activities, *P. gingivalis* relative abundance, and MDC and MIP-1α concentrations) over time (baseline, weeks 2, 6, and 12). BOP was included in the model as a time-varying covariate. Study participants were also categorized as “high” (equal or over the median value) or “low” (below the median value) based on their salivary *P. gingivalis* gingipain activity, total protease activity, and *P. gingivalis* relative abundance, separately. To analyze the difference in BOP, PI, MIP-1α, and MCP levels between the groups of high and low gingipain activity, high and low total protease activity, and high and low *P. gingivalis* relative abundance over time, a general linear model for repeated measures analysis was used. *p* value of < 0.05 was accepted as statistically significant.
